# Diagnostic Accuracy of Loopamp *Trypanosoma brucei* Detection Kit for Diagnosis of Human African Trypanosomiasis in Clinical Samples

**DOI:** 10.1371/journal.pntd.0002504

**Published:** 2013-10-17

**Authors:** Patrick Mitashi, Epco Hasker, Dieudonné Mumba Ngoyi, Pati Patient Pyana, Veerle Lejon, Wim Van der Veken, Pascal Lutumba, Philippe Büscher, Marleen Boelaert, Stijn Deborggraeve

**Affiliations:** 1 Institute of Tropical Medicine, Antwerp, Belgium; 2 Faculty of Medicine, Kinshasa University, Kinshasa, Democratic Republic of the Congo; 3 Institut National de Recherche Biomédicale, Kinshasa, Democratic Republic of the Congo; 4 Belgian Development Agency, Kinshasa, Democratic Republic of the Congo; Foundation for Innovative New Diagnostics (FIND), Switzerland

## Abstract

**Background:**

Molecular methods have great potential for sensitive parasite detection in the diagnosis of human African trypanosomiasis (HAT), but the requirements in terms of laboratory infrastructure limit their use to reference centres. A recently developed assay detects the *Trypanozoon* repetitive insertion mobile element (RIME) DNA under isothermal amplification conditions and has been transformed into a ready-to-use kit format, the Loopamp *Trypanosoma brucei*. In this study, we have evaluated the diagnostic performance of the Loopamp *Trypanosoma brucei* assay (hereafter called LAMP) in confirmed *T.b. gambiense* HAT patients, HAT suspects and healthy endemic controls from the Democratic Republic of the Congo (DRC).

**Methodology/Principal findings:**

142 *T.b. gambiense* HAT patients, 111 healthy endemic controls and 97 HAT suspects with unconfirmed status were included in this retrospective evaluation. Reference standard tests were parasite detection in blood, lymph or cerebrospinal fluid. Archived DNA from blood of all study participants was analysed in duplicate with LAMP. Sensitivity of LAMP in parasitologically confirmed cases was 87.3% (95% CI 80.9–91.8%) in the first run and 93.0% (95% CI 87.5–96.1%) in the second run. Specificity in healthy controls was 92.8% (95% CI 86.4–96.3%) in the first run and 96.4% (95% CI 91.1–98.6%) in the second run. Reproducibility was excellent with a kappa value of 0.81.

**Conclusions/Significance:**

In this laboratory-based study, the Loopamp *Trypanosoma brucei* Detection Kit showed good diagnostic accuracy and excellent reproducibility. Further studies are needed to assess the feasibility of its routine use for diagnosis of HAT under field conditions.

## Author Summary

Diagnosis and effective treatment are cornerstones in the control of human African trypanosomiasis (HAT). Molecular tools such as the polymerase chain reaction (PCR) detect the parasite's DNA and are generally very sensitive and specific. However, PCR is not applicable in field settings because it requires a laboratory infrastructure and sophisticated equipment. A recently developed loop-mediated isothermal amplification (LAMP) has emerged as a simpler alternative to conventional molecular methods for the diagnosis of HAT. The test has been transformed into a diagnostic kit for qualitative detection of the parasite's DNA in clinical specimens, the Loopamp *Trypanosoma brucei* Detection Kit. In this study, we evaluated this kit in laboratory conditions on DNA extracted from blood samples of 142 patients, 97 suspects and 111 healthy endemic controls in the Democratic Republic of the Congo. The test showed good diagnostic accuracy and excellent reproducibility. Given the practical advantages of LAMP over conventional nucleic acid methods these are promising results. Further studies are needed to assess the test's accuracy and feasibility in field conditions.

## Introduction

Human African trypanosomiasis (HAT) is a protozoan disease caused by the *Trypanosoma brucei* species, which are cyclically transmitted by tsetse flies. Two subspecies are pathogenic to man: *Trypanosoma brucei (T.b.) gambiense* in central and western Africa, and *Trypanosoma brucei rhodesiense* in east and southern Africa [1]. Currently, less than 10 000 cases per year are reported by the World Health Organization, of which over 70% occur in the Democratic Republic of Congo (DRC) [2].

Diagnostic algorithms for *T.b. gambiense* HAT generally start using the Card Agglutination Test for Trypanosomiasis (CATT) as initial screening for the presence of antibodies. Those testing positive in CATT are then subjected to parasitological tests for confirmation of the infection [3]. Parasitological confirmation relies on the microscopic search for parasites either in lymph, blood or cerebrospinal fluid (CSF). The most sensitive method is based on the mini-anion exchange centrifugation technique (mAECT), yielding an analytical sensitivity of <50 parasites per mL of blood [4], [5]. However, given the low parasitemia associated with *T.b.* g*ambiense* infection, some truly infected individuals remain negative in the mAECT.

Because of the limited sensitivity of parasitological confirmation tests, molecular methods have been developed [6], [7] and they generally show high sensitivity and specificity [7]. They can be performed on various specimen types such as whole blood, blood stored on filter paper and CSF. However, the need for laboratory instruments for nucleic acid extraction, amplification and visualization are obstacles to their application in clinical settings in HAT endemic areas [8]. Isothermal reactions such as nucleic acid sequence-based amplification (NASBA) and loop mediated isothermal amplification (LAMP) have recently been developed for the diagnosis of HAT [7], [9]. In contrast to PCR, they do not require thermocyclers and amplification can be conducted in a heating block or a hot water bath. A potential advantage of NASBA is that it targets RNA and thus might have greater utility as a test of cure compared with DNA-targeting molecular tests [6]. However, NASBA is not yet ready to be used under field conditions due to the complexity of RNA purification [6]. Instead of RNA, LAMP amplifies DNA that is less prone to damage during transport and storage of samples and during extraction. Sets of specific inner and outer primers are needed for autocycling strand displacement DNA synthesis by the *Bst* DNA polymerase at a temperature between 60–65°C. The results can be interpreted by several detection formats, such as turbidity, fluorescent DNA intercalating dyes, fluorescent hybridisation probes and oligochromatography [9], [10].

There are published reports on two LAMP assays for *Trypanozoon* DNA. One assay targets the single copy paraflagellar rod protein A (PfrA) gene and the second is based on the repetitive insertion mobile element (RIME) [9]. Recently, the latter has been transformed into a commercially available kit, the Loopamp *Trypanosoma brucei* kit (Eiken Chemical Co LTD, Japan in collaboration with FIND, Geneva, Switzerland) [9]. Ready-to-use reaction tubes are provided with the reagents dried down in the caps of the tubes, together with negative and positive controls. LAMP showed great promise with purified DNA and with trypanosome-spiked blood but has not been yet evaluated on specimens from HAT patients and controls. We here present the data from the first diagnostic evaluation of the commercial LAMP kit on DNA extracted from blood of 142 *gambiense* HAT patients, 97 *gambiense* HAT suspects and 111 healthy endemic controls from the DRC.

## Methods

### Ethical clearance

All samples analysed in this study were collected within the framework of two earlier diagnostic studies for HAT, PARAHAT and HAT-PolyB. Both studies were approved by the ethical committees of the University Hospital in Antwerp (registration numbers ITG09415684 and B30020108363, respectively) and the Ministry of Health of the D.R Congo (registration numbers M-D/226/2010 and M-D/179/2010, respectively). The ethical committees approved extended use of the samples in further HAT diagnostic studies. Written informed consent was obtained from all study participants and all samples were anonymized.

### Characteristics of study samples

For this retrospective evaluation we used DNA extracts from blood of study participants recruited consecutively in 2010 in Bandundu, the most HAT endemic province in DRC [11]. From all participants testing positive on CATT whole blood, the CATT was repeated with sequential plasma dilutions and the end titer was recorded [3]. For diagnostic purposes all were subjected to parasitological confirmation, irrespective of CATT results. Trypanosomes were detected by examination of lymph node aspirate (in subjects with swollen cervical nodes) or by blood examination (all study subjects) with the capillary centrifugation technique (CTC) [12], mAECT on whole blood [8], and mAECT on buffy coat [5]. For patients with parasites detected in the lymph or blood, or with a plasma CATT end titer ≥1∶8 a lumbar puncture was done. Parasite detection in CSF was performed with the single modified centrifugation technique [4]. DNA was extracted from blood with the Maxwell® 16 Blood DNA Purification robot (Promega Corporation, Madison, WI,USA) from 200 µL blood stabilised in an equal volume of DNA stabilising GE buffer (6 M guanidium, 0.2 M EDTA, pH = 7.5). Final DNA extraction volumes were 300 µL and extracts were stored at −20°C. Time between DNA extraction and LAMP testing was 1.5 to 2 years. All blood samples were also analysed with a *Trypanozoon*-specific 18S rDNA PCR in duplicate [13]. This PCR amplifies a 120 bp DNA sequence of the *Trypanozoon* 18S rRNA gene and the amplified product is visualized using conventional electrophoresis in agarose gels and ethidium bromide staining. All PCR testing was done in duplicate at the Institute of Tropical Medicine in Antwerp.

Participants were considered as HAT patients if parasites were detected by any parasitological method in any blood or lymph or CSF sample. Healthy endemic controls were recruited during active screening in the villages [14]. Healthy endemic controls are individuals presenting themselves for CATT screening but with no clinical symptoms of HAT, no previous history of HAT and negative results in CATT whole blood, trypanolysis and mAECT. Individuals with suggestive clinical findings, a positive CATT (cut-off titer ≥1∶4) and positive trypanolysis test that were not confirmed as cases on parasitological testing and who had no previous history of HAT, were classified as HAT suspects. Altogether, frozen DNA from blood of 350 study participants were tested by LAMP: 142 from confirmed HAT patients, 97 from HAT suspects and 111 from healthy endemic controls. In the confirmed HAT patient group, standard tests showed parasites in the blood in 131 cases while in 5 and 6 cases parasites were only found in the CSF and lymph respectively.

### Index test: Loopamp *Trypanosoma brucei* Detection Kit

The Loopamp *Trypanosoma brucei* Detection Kit (Eiken Chemical,Taito-ku, Tokyo, Japan) was applied in duplicate on the DNA extracts by one of the authors (PM), a trained clinical microbiologist, who was blinded to the disease status of the samples. The test was performed at ITM Antwerp according to the product insert. Briefly, the dried reagents in the tube were reconstituted in a 25 µl reaction solution, containing 3 µl template DNA and 22 µl negative control buffer, and immediately placed in the LAMP incubator (LF-160 incubator, Eiken Chemical co,Taito-ku, Tokyo, Japan). LAMP amplified *Trypanosoma brucei* DNA was visualised using the provided UV-LED device. Amplified DNA emits green fluorescence while there is no fluorescence in negative samples. The provided positive and negative controls were taken in each run (14 tests) to validate the test results.

### Data analysis

Sensitivity and specificity values and their 95% confidence intervals were calculated for the LAMP in the confirmed HAT patients and in healthy endemic controls, respectively. The sensitivity was defined as the proportion of confirmed HAT patients who are positive by the index tests and specificity as the proportion of healthy endemic controls who are negative by the index test. Each DNA extract was tested in duplicate by LAMP. Agreement between LAMP and PCR and reproducibility of LAMP were assessed on all specimens (patients, suspects, controls) with Cohen's Kappa and interpreted following the grading system described by Landis and Koch (1977) [15]. Data were analysed in Stata, version 11.1 (StataCorp, College Station, Lakeway, Texas, USA).

## Results

### Diagnostic accuracy

Of the 142 HAT patients, 132 and 124 were LAMP positive in respectively the first and second run, corresponding with sensitivities of 93.0% (95% CI: 87.5%–96.1%) and 87.3% (95% CI : 80.9–91.8), respectively (table 1). Of the 11 patients with trypanosomes detected only in lymph or in CSF, 7 were positive in both LAMP runs on blood. Of the 97 HAT suspects, 6 were positive in both replicates of LAMP, 8 and 20 were positive in the first and second replicate, respectively. Of 111 healthy endemic controls, 4 tested positive twice with LAMP and 4 tested positive only once. Specificity estimates range from 92.8% (95% CI 86.4–96.3%) to 96.4% (95% CI 91.1%–98.6%). Sensitivities and specificities of PCR were in the same range as LAMP with overlapping confidence intervals (table 1).

**Table 1:** Sensitivities and specificities of replicate RIME LAMP and 18S PCR on the blood of HAT patients and healthy endemic controls.

		HAT patients (n = 142)		Healthy endemic controls (n = 111)	
Test		Positive results	Sensitivity% (95% CI)	Positive results	Specificity% (95% CI)
LAMP	Run 1	132	93.0 (87.5–96.1)	4	96.4 (91.1–98.6)
	Run 2	124	87.3 (80.9–91.8)	8	92.8 (86.4–96.3)
PCR	Run 1	124	87.3 (80.9–91.8)	4	96.4 (91.1–98.6)
	Run 2	128	90.1 (84.1–94.0)	3	97.3 (92.3–99.0)

Note. n: number of specimens, CI: confidence interval.

### Agreement between molecular methods

Assessed on all participants (patients, suspects and healthy controls), agreement between the two LAMP replicates was excellent with a kappa value of 0.81 (95% CI: 0.71–0.92) (table 2), which is in the same range as the PCR replicates (kappa value = 0.82, 95% CI: 0.72–0.92). Agreement between the first replicate of LAMP and the 18S PCR was also excellent with a kappa value of 0.82 (95% CI: 0.72–0.93). Kappa values of LAMP replicates were lower in the subgroups but in the same range as for PCR and with overlapping confidence intervals (table 2).

**Table 2:** Agreement between the two LAMP replicates and between LAMP and PCR.

Group	LAMP vs LAMP	PCR vs PCR	LAMP vs PCR
	Kappa value (95% CI)	Kappa value (95% CI)	Kappa value (95% CI)
All study participants	0.81 (0.71–0.92)	0.82 (0.72–0.92)	0.82 (0.72–0.92)
HAT patients	0.65 (0.48–0.82)	0.58 (0.42–0.74)	0.61 (0.45–0.77)
HAT suspects	0.35 (0.18–0.52)	0.50 (0.30–0.70)	0.39 (0.22–0.56)
Healthy endemic controls	0.53 (0.37–0.69)	0.56 (0.38–0.74)	0.48 (0.3–0.66)

## Discussion

In this diagnostic accuracy study, the LAMP showed a sensitivity of 87.3% and 93.0% in the two testing runs. Specificity was 92.8% and 96.4%, with a lowest lower limit of the 95% confidence interval of 86.4%. Agreement between LAMP replicates as well as between LAMP and PCR was excellent with kappa values above 0.8.

The sensitivity of the commercial LAMP kit tested here was equivalent to that of the 18S PCR test, which showed a sensitivity between 87.3% (95% CI: 80.9–91.8) and 90.1% (95% CI: 84.1–94.0) on the same DNA extracts. This is in line with the observation that both tests showed identical analytical sensitivities of 100 parasites per mL of blood in a head-to-head comparison using experimentally prepared blood samples (data not shown). While the LAMP detects the RIME DNA elements (500 copies per haploid genome) [16], the PCR targets the 18S rRNA gene (10–100 copies) [17]. In the 11 confirmed HAT patients with parasites only detected in the lymph or CSF, 7 were positive in both LAMP runs. In contrast, we also observed 5 false negative LAMP results in mAECT positive patients. In the HAT suspects, who could not be confirmed by the parasitological methods, we observed particularly poor agreement between the two LAMP repetitions (kappa = 0.35). These discordances are probably due to the fact that the target DNA concentration in such samples is at the detection limit of the test. If LAMP is to be used to confirm non-confirmed HAT suspects, testing multiple samples from the same patient may increase its sensitivity.

The specificity of the LAMP kit was in the same range as the 18S PCR, which showed a specificity between 96.4% (95% CI 91.1–98.6%) and 97.3% (95% CI 92.3–99.1%) on the same samples. The LAMP was twice positive in 4 of the 111 healthy endemic controls. Three of these LAMP positive controls were at least one time also positive by PCR. Some positive healthy endemic controls may actually be infected with *T.b. gambiense* because the parasitological confirmation algorithm using mAECT is not 100% sensitive, and this may lead to an underestimation of the specificity of the index tests. Another possible reason may be the absence of the LiTat 1.3 variable surface glycoprotein (VSG), which is the antigen used in the CATT and the trypanolysis test, in some strains of *T.b. gambiense* [18], [19]. In addition, low antibody titers may be present in early or latent infections [20]. However, false positive LAMP results due to non-specific amplification reactions cannot be excluded. Since the LAMP detects the RIME DNA of all *Trypanozoon*, a transient human infection with *T.b. brucei* could also have led to a positive test result [21]. The recently developed LAMP assay that targets the *T.b. gambiense* specific glycoprotein (TgsGP) gene [16] can exclude an infection with other *Trypanozoon* and thus may be more specific. However, in the same publication the authors showed that the diagnostic sensitivity of the TgsGP LAMP is lower than the sensitivity of the RIME LAMP.

Reproducibility of LAMP was excellent and as good as that of PCR, with kappa values of 0.81 and 0.82 respectively when all samples were considered. Within the sub groups lower kappa values were observed, which is due to the fact that in these more homogenous groups the expected agreements were much higher. Values observed within the groups were in the same ranges for LAMP and PCR. The LAMP-amplified DNA is visualised by a UV-LED device attached to the LF-160 incubator. This single-tube and easy read-out avoids the risk for sample contamination due to post-amplification manipulations. Another advantage is that Loopamp *Trypanosoma brucei* Detection Kit is thermostable at 30°C which greatly enhances the feasibility of use in peripheral health facilities in tropical countries. Although in the present study LAMP was performed on DNA extracted with the Maxwell® DNA Purification robot, simplified DNA extraction methods that are compatible with LAMP are currently under development. The requirement of electrical power supply to operate the incubator for the amplification step constitutes a potential drawback for use in remote health facilities, even if it can be circumvented by using an alternative power source such as an electrical generator and/or a photovoltaic panel.

In recent years there has been a sharp decline in HAT prevalence in most of the endemic countries and the classical case finding approach by mobile screening units is becoming less cost-effective. There is thus an urgent need to consider alternative ways of surveillance and case detection, and the LAMP technology could play a role [22]. Though still more complicated than the parasitological methods, LAMP is feasible for use at the level of a district hospital laboratory and could be useful as part of a testing algorithm for samples collected at more peripheral levels. LAMP can be applied on samples collected elsewhere without the need to be processed the same day. Either CATT or one of the newly developed rapid tests [23] can be used to screen suspects for HAT at health center or at village level; LAMP can then be used in a more centrally located laboratory as a second step in the diagnostic algorithm. LAMP data for serologically positive individuals who remained negative in parasitological testing may be particularly informative. However, future research should determine if HAT suspects with positive LAMP testing need further diagnostic work-up before being put on treatment and if the detection of LAMP positive individuals from the same geographical origin should be a trigger for intensified surveillance efforts. Although we feel that LAMP is best suited for use in central laboratories, the feasibility and cost-effectiveness of including LAMP in the screening process by the mobile teams may be determined in specific evaluation studies but should also take into account the test specimen preparation prior to the LAMP itself.

In conclusion, the study shows that the LAMP has similar diagnostic accuracy as the 18S rDNA PCR and can replace PCR for accurate and simplified detection of *Trypanozoon* DNA in clinical specimens. LAMP may have an important role to play in disease surveillance. However, one should note that the specificity of LAMP is not 100%, that HAT treatment is complex and toxic, and that the positive predictive value of tests in low incidence settings is low. Based on this study we cannot yet recommend initiating treatment of patients based on LAMP results; further evidence from prospective clinical studies under field conditions is needed, as well as cost-effectiveness analysis of competing algorithms. Feasibility studies of LAMP are currently conducted in the D.R. Congo (http://www.finddiagnostics.org/programs/hat-ond/hat/molecular_diagnosis.html).

## Supporting Information

Checklist S1 STARD checklist showing that all essential elements of a diagnostic evaluation study are included in the manuscript.
(PDF)

Figure S1 STARD flowchart describing the design of the study and the flow of the participants.
(PDF)

Table S1 Excel spreadsheet with the raw data of the reference tests and index test in the study population.
(XLS)
