# Supplementary figures and images for: Diagnostic Accuracy of Loopamp Trypanosoma brucei Detection Kit for Diagnosis of Human African Trypanosomiasis in Clinical Samples
Source: PLoS Negl Trop Dis. 2013 Oct 17;7(10):e2504. doi: 10.1371/journal.pntd.0002504 (PMC3798548; doi:10.1371/journal.pntd.0002504)

General example

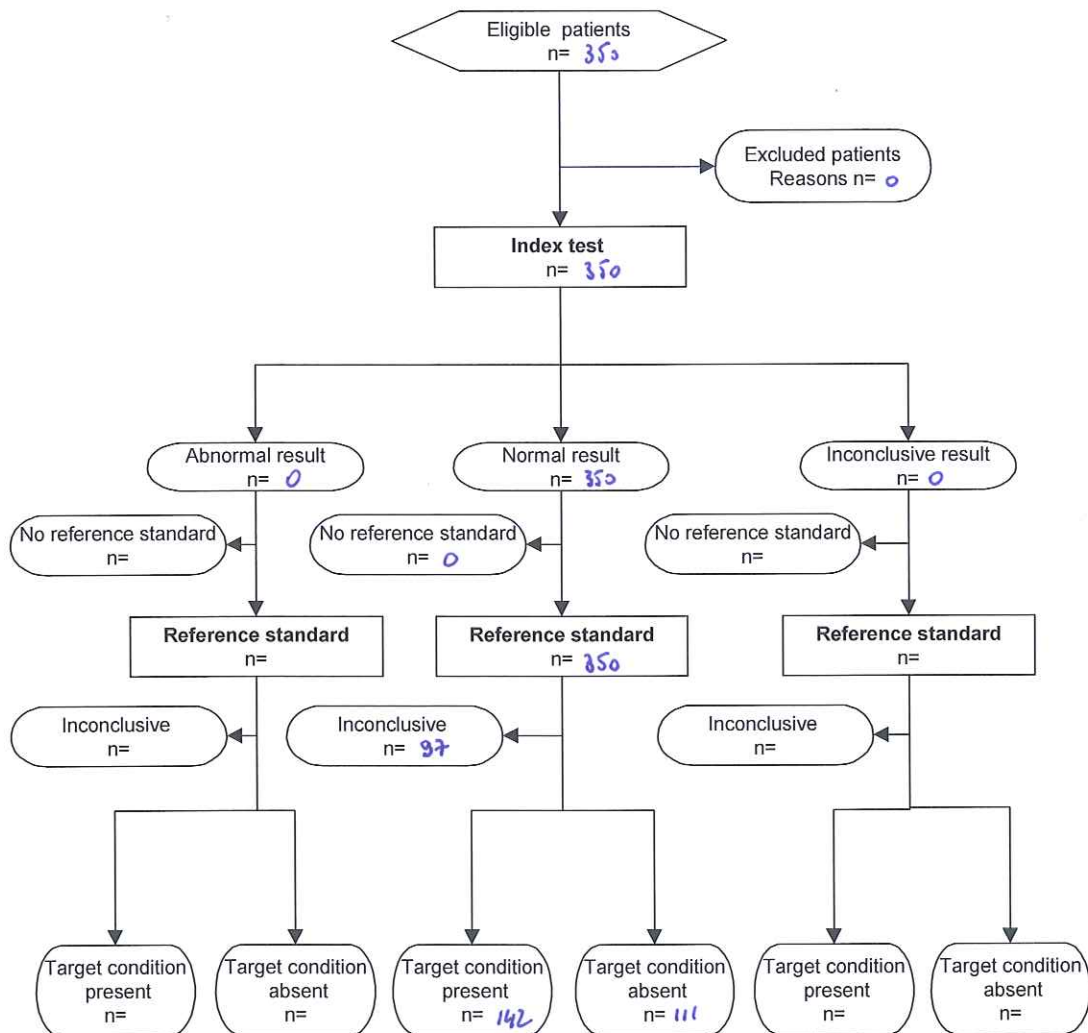

Supplement: Figure S1 — STARD flowchart describing the design of the study and the flow of the participants. (PDF) [file pntd.0002504.s002.pdf]
